# USING FOUND POETRY TO EXPLORE SEXUAL AND GENDER MINORITY COUPLES' EXPERIENCES FACING CANCER

**DOI:** 10.1093/geroni/igac059.541

**Published:** 2022-12-20

**Authors:** Sara Bybee, Kristin Cloyes, Kathi Mooney, Katherine Supiano, Brian Baucom, Lee Ellington

**Affiliations:** University of Utah, Salt Lake City, Utah, United States; Oregon Health & Science University, Portland, Oregon, United States; University of Utah College of Nursing, Salt Lake City, Utah, United States; University of Utah, Salt Lake City, Utah, United States; University of Utah Department of Psychology, Salt Lake City, Utah, United States; University of Utah and Huntsman Cancer Institute, Salt Lake City, Utah, United States

## Abstract

This study explored how relationships of sexual and gender minority (SGM) couples change through the cancer experience. Twelve couples (N = 24) completed surveys assessing demographics and dyadic semi-structured interviews. Thematic analysis was used to analyze interview transcripts. Participants had been together for 19.1 years on average (SD = 9.9, R = 9-44) and commonly described dyadic strength and durability as a result of cancer. Using in-vivo language extracted from the theme dyadic strength and durability, a found poem was constructed depicting how couples saw themselves as two-person teams united against any external stressors. When SGM couples experienced cancer together, it resulted in feeling closer to one another, like they could handle anything that came their way, and assured them that they would stay together regardless of any future hardships experienced. Creative qualitative methods revealed SGM couples facing cancer felt like unyielding, impenetrable, eternal duos with which to be contended.

